# A panel of three oxidative stress-related genes predicts overall survival in ovarian cancer patients received platinum-based chemotherapy

**DOI:** 10.18632/aging.101473

**Published:** 2018-06-17

**Authors:** Jin Zhang, Lixiao Yang, Xiaohong Xiang, Zhuoying Li, Kai Qu, Ke Li

**Affiliations:** 1 Department of Clinical Laboratory, Liaocheng People’s Hospital, Taishan Medical College, Liaocheng 252000, Shandong Province, China; 2 Department of Obstetrics and Gynecology, Liaocheng People’s Hospital, Taishan Medical College, Liaocheng 252000, Shandong Province, China; 3 Department of Hepatobiliary Surgery, The First Affiliated Hospital of Xi’an Jiaotong University, Xi’an 710061, Shaanxi Province, China; 4 Department of Breast Surgery, The First Affiliated Hospital of Xi’an Jiaotong University, Xi’an 710061, Shaanxi Province, China; 5 Department of Central Laboratory, Liaocheng People’s Hospital, Taishan Medical College, Liaocheng 252000, Shandong Province, China; * Equal contribution

**Keywords:** oxidative stress, prognosis, ovarian cancer, platinum, chemotherapy

## Abstract

Ovarian cancer yields the highest mortality rate of all lethal gynecologic cancers, and the prognosis is unsatisfactory with the major obstacle in resistance to chemotherapy. The generation of reactive oxygen species (ROS) in tumor tissues was associated with chemotherapeutic effectiveness by mediating cellular longevity. In this study, we screened the prognostic values of oxidative stress-related genes in ovarian cancer patients received platinum-based chemotherapy, and conducted a prognostic gene signature composing of three genes, *TXNRD1*, *GLA* and *GSTZ1*. This three-gene signature was significantly associated with overall survival (OS), but not progression-free survival (PFS), in both training (n=276) and validation cohorts (n=230). Interestingly, we found that the prognostic value of three-gene signature was reinforced in platinum-sensitive patients. Subgroup analysis further suggested that patients with elder age, higher pathological grades and advanced tumor stages in low-risk score group could benefit from platinum-based chemotherapy. Functional analysis showed that the inactivation of several signaling pathways, including cell cycle, insulin-like growth factor 1 (IGF1) /mTOR and Fas pathways, was affected by three genes. Collectively, our results provided evidence that a panel of three oxidative stress-related gene signature had prognostic values for ovarian cancer patients received platinum-based chemotherapy.

## Introduction

Ovarian cancer, as one of the most lethal malignancies among females, had approximately 238,700 newly diagnosed cases each year worldwide [1,2]. Due to its vague symptoms and lack of effective biomarkers, most patients were usually diagnosed at advanced stages [1,3–6]. Despite recent advancements in therapies, the prognosis of ovarian cancer is still unsatisfactory with the major obstacle in resistance to standard platinum-based chemotherapy. So far, combination of cyto-reductive surgery and post-operative chemotherapy is the current standard treatment for advanced ovarian cancer. However, more than 70% patients developed resistance to the platinum-based chemotherapy after surgery within six months [7–9]. The clinical characteristics, such as histologic type, tumor grade, debulking status and CA-125 levels, did not achieve satisfied prognostic values for ovarian cancer patients [10]. Therefore, it is essential to explore promising prognostic biomarkers in ovarian cancer patients.

During the past decade, great efforts have been made to explore the molecular mechanisms involved in the response to platinum-based chemotherapy in ovarian cancer patients. It has been well-demonstrated that chemotherapy-induced oxidative stress was associated with chemotherapeutic effectiveness [11]. Mechanistic investigations showed that the generation of reactive oxygen species (ROS) caused genomic instability in tumor cells and promoted cellular apoptosis, senescence or autophagy [12]. Thus, the intracellular balance of oxidants and antioxidants contributed to the therapeutic effectiveness in ovarian cancer patients received platinum-based chemotherapy. Indeed, several oxidative stress-related genes, such as *ARHGEF6* [13] and *ALDH1* [14], have been reported to be related to chemo-resistance in ovarian cancer. However, effective molecular biomarkers accurately predicting clinical prognosis in ovarian cancer patients received platinum-based chemotherapy have not yet been thoroughly explored.

In this study, we performed comprehensive investigations to identify the prognostic gene signature from 99 oxidative stress-related genes. Using cox regression analysis, we developed a three-gene prognostic signature consisting of *TXNRD1*, *GLA* and *GSTZ1*, and validated this model in another independent cohort. Additionally, we also performed bioinformatic analysis to explore the potential molecular mechanisms underlying the different clinical outcomes of ovarian cancer patients.

## RESULTS

### Construction of prognostic model based on oxidative stress-related genes in the training group

**Figure 1:**
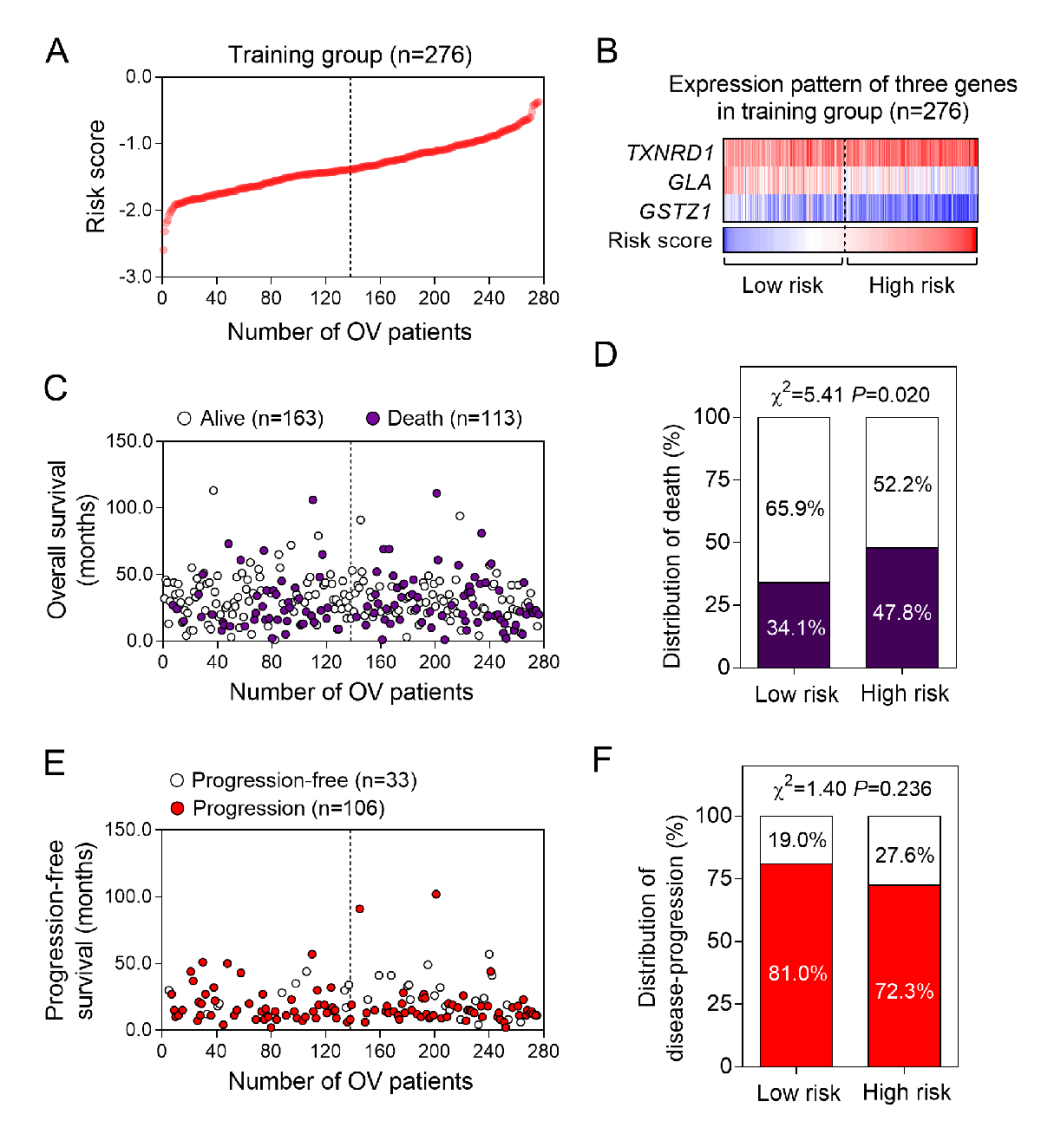
**The three-gene signature-focused risk score in prognosis of overall survival in the validation group.** (**A**) The three gene-based risk score distribution. (**B**) The heatmap of the expression of three genes. (**C**) Patients’ overall survival status in training group. (**D**) The mortality rate in low- and high-risk score groups. (**E**) Patients’ progression-free survival status in training group. (**F**) The recurrence rate in low- and high-risk score groups.

Firstly, we employed 276 ovarian cancer patients to construct the prognostic model by using oxidative stress-related genes. All oxidative stress-related genes were listed in Table S1. By subjecting the genes expression data to Cox regression analysis, we identified a panel of three oxidative stress-related genes consisting of *TXNRD1, GLA and GSTZ1,* which were strongly correlated with patients’ overall survival (Table 1, *P<*0.05). We then calculated the risk score for each patient in the training group by using the risk score formula. Using the median risk score as cut-off value, the patients in the training group were divided into low (n = 138) and high (n = 138) risk score subgroups (Figure 1A). As shown in Figure 1B, the expression patterns showed that the patients in high risk score group had higher *TXNRD1* expression and lower *GLA and GSTZ1* expression.

**Table 1:** Three-genes signature associated with the OS of ovarian cancer patients received platinum-based chemotherapy.

**Symbol**	**GeneBank**	**HR**	**95%CI of HR**	**Coefficient**	***P*-value**
*GLA*	NM_000169	0.69	0.49-0.96	-0.38	0.027
*GSTZ1*	NM_001513	0.70	0.51-0.97	-0.36	0.033
*TXNRD1*	NM_003330	1.59	1.02-2.47	0.46	0.040

**Figure 2:**
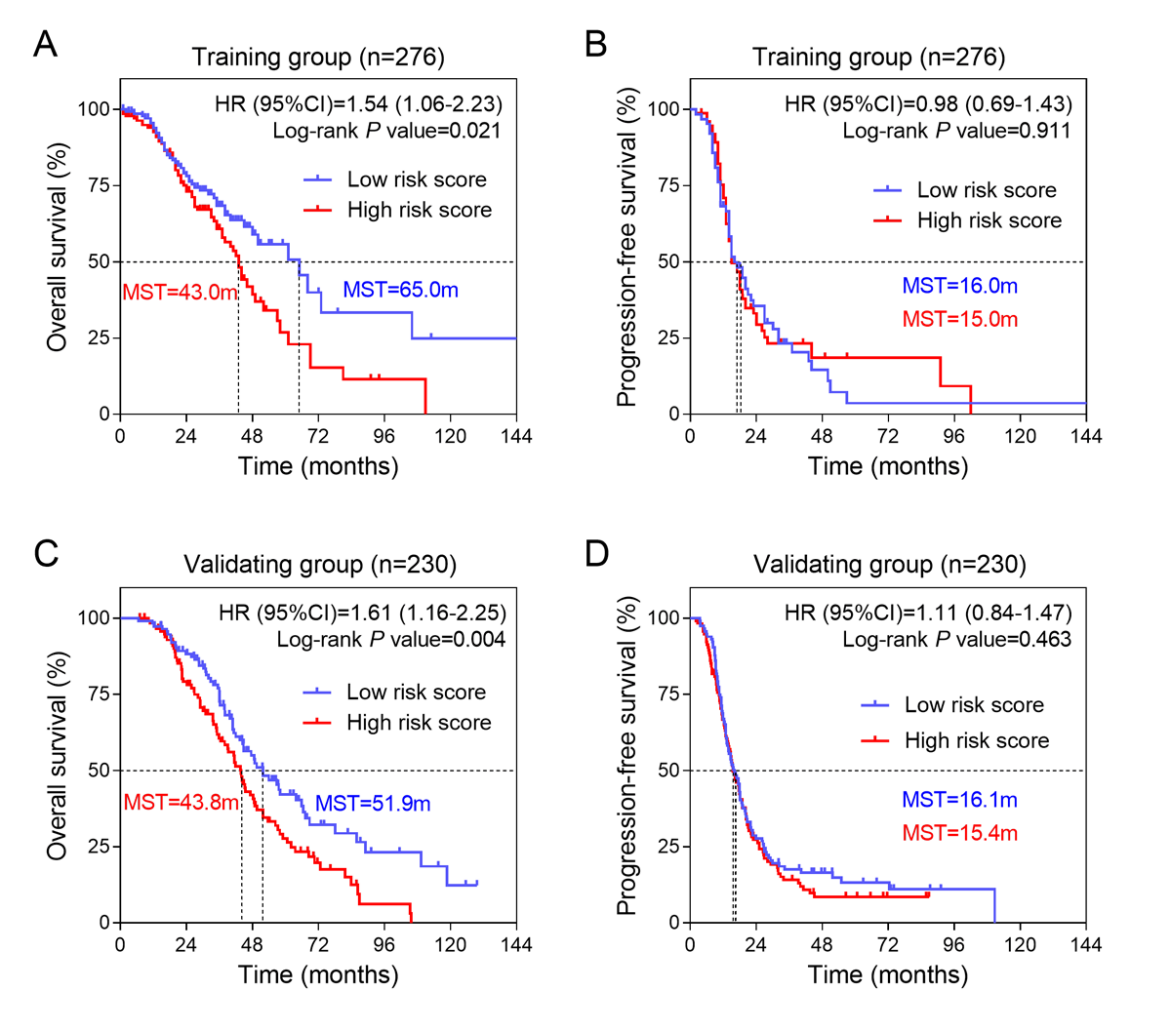
**The association between three-gene signature and survival in training and validation groups.** (**A**) Kaplan-Meier survival curves were plotted to estimate the overall survival probabilities for the low-risk versus high-risk group in training group (n=276). (**B**) Progression-free survival was estimated by Kaplan-Meier curves in training group (n=276). (**C**) Overall survival and (**D**) progression-free survival were estimated in validation group (n=230).

Next, we analyzed the differences of clinical outcomes between high and low risk score groups (Figure 1C). Our data suggested that the mortality rate in high risk score group was significantly higher than low risk score group (Figure 1D, *P*=0.020). Moreover, we also analyzed the disease progression status in 139 patients who had tumor progression information (Figure 1E). Unexpectedly, we found there is no differences of tumor progression status between high and low risk score groups (Figure 1F). To further explore the association between the three-gene signature and survival, we performed the Kaplan-Meier curves to estimate the MST between two groups. As expected, patients in the high risk score group had significantly shorter overall survival time (MST=43.0 months) than those in the low risk score group (MST=65.0 months) [HR (95%CI) =1.54 (1.06-2.23); log-rank *P* value=0.021] (Figure 2A). However, we find no significance in progression-free survival between the high and low risk score groups [15.0 months vs 16.0 months; HR(95%CI) =0.98 (0.69-1.43); log-rank *P* value=0.911] (Figure 2B).

### Validation of the three-gene signature for survival prediction in the validation group

To validate our findings, we calculated the risk score for ovarian cancer patients in an independent validation group (n = 230) using the same formula. Because the gene expression profiles in validation group were based on RNA-sequencing platform, which was different from the training group (Affymetrix Human Genome U133 Plus 2.0 platform), we did not use same cut-off value as the training group, but selected the median value in training group as the cut off. The patients from the validation group were divided into low and high risk score groups and then subjected to survival comparison. Similar to the findings obtained from the training group, patients in the high risk score group had shorter overall survival time than patients in the low risk score group [43.8 months vs 51.9 months; HR(95%CI) =1.61(1.16-2.25); log-rank *P* value=0.004] (Figure 2C). Similarly, there was no significance in progression-free survival between the two groups [15.4 months vs 16.1 months; HR (95%CI) =1.11 (0.87-1.47); log-rank *P* value=0.463] (Figure 2D).

### Prognostic values of three-gene signature for patients with different therapeutic response in validation group

**Figure 3:**
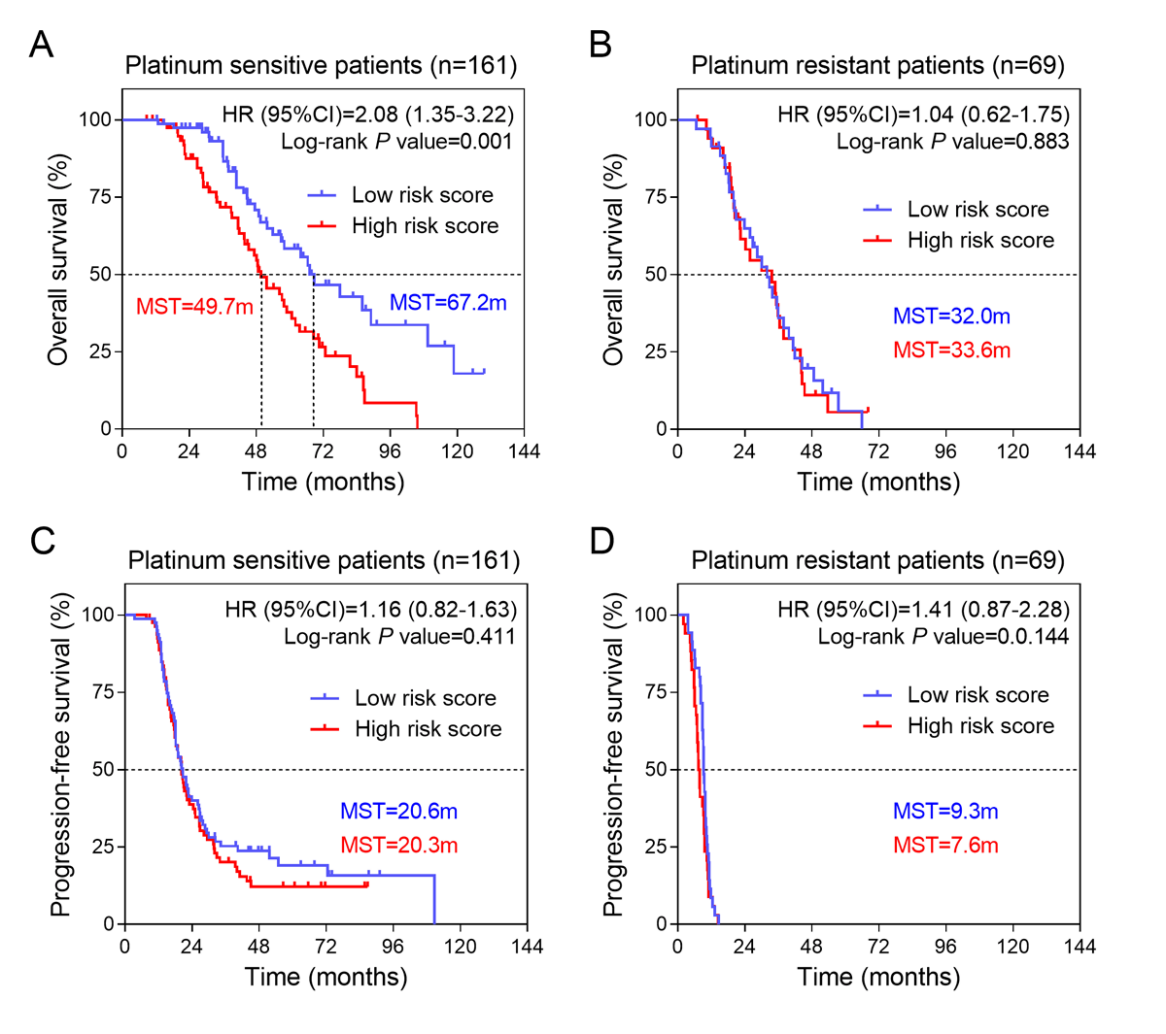
**Kaplan-Meier estimates of the survival of patients with different platinum response in training group.** (**A**) Kaplan-Meier survival curves were plotted to estimate the overall survival for platinum sensitive patients in validation group (n=161). (**B**) Kaplan-Meier survival curves were plotted to estimate the overall survival for platinum resistant patients in validation group (n=69). Progression-free survival was estimated by Kaplan-Meier curves for (**C**) platinum sensitive and (**D**) platinum resistant patients in training group.

To further explore the prognostic values of three-gene signature for the platinum sensitive and resistant patients, we picked up platinum sensitive patients (n = 161) and resistant patients (n = 69) from the validation group and conducted Kaplan-Meier curves separately. Interestingly, we found that the three-gene signature had a high accuracy to predict overall survival only in the platinum sensitive patients [HR (95%CI) =2.08 (1.35-3.22); log-rank *P* value=0.001] (Figure 3A). There was no significant association between three-gene signature and overall survival in platinum resistant patients [HR (95%CI) =1.04 (0.62-1.75), log-rank *P* value=0.883] (Figure 3B). In addition, three-gene signature was found to be insignificantly associated with progression-free survival both in the platinum sensitive (Figure 3C) and platinum resistant patients (Figure 3D).

### Subgroup analysis of three-gene expression signature in predicting overall survival of platinum-sensitive patients

**Figure 4:**
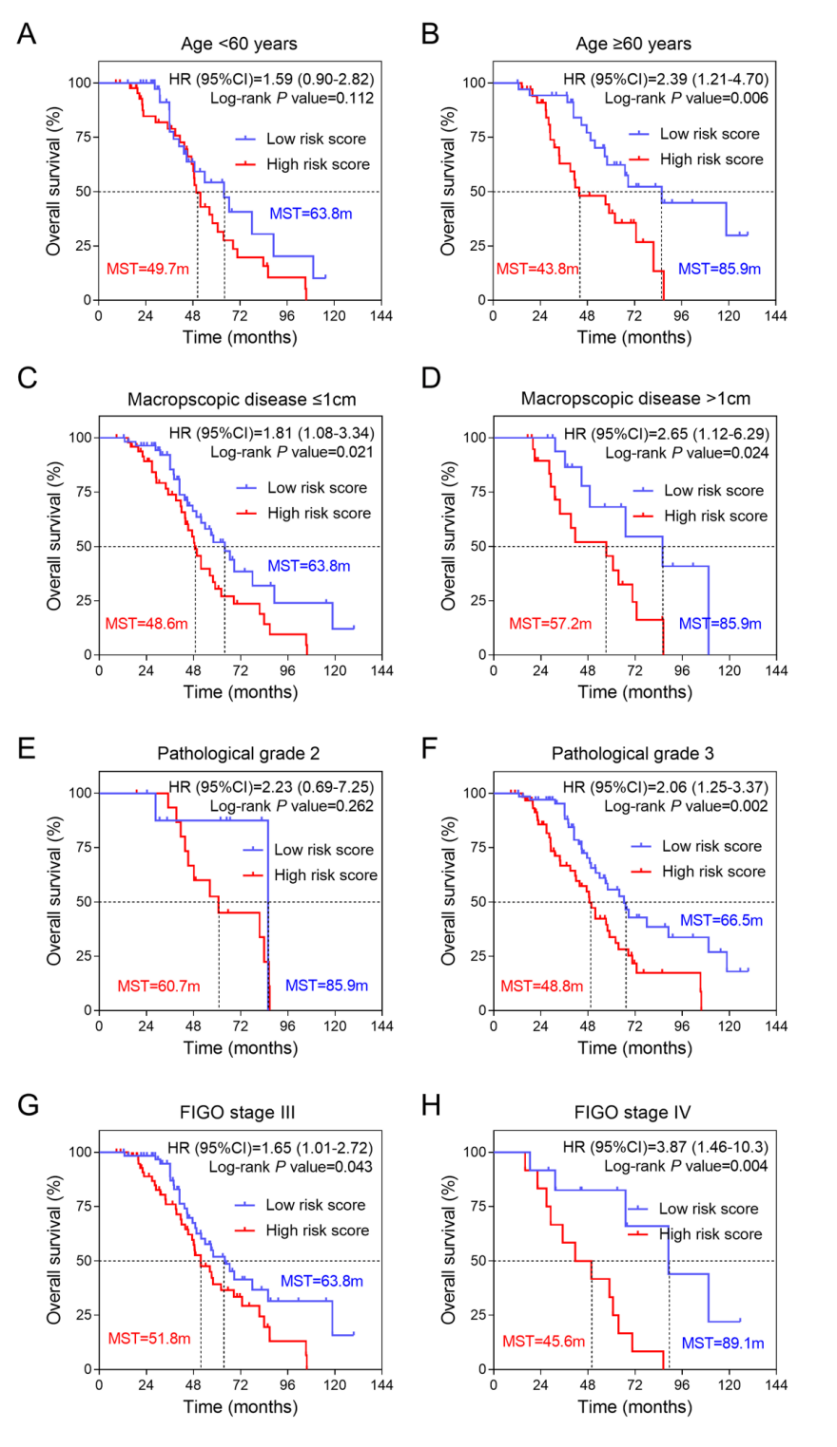
**Effects of SAMR1 and SAMP8 mice fecal microbiota transplant on behavior in pseudo germ-free mice.** (**A**) Kaplan-Meier curves for younger patients (age<60 years). (**B**) Kaplan-Meier curves for older patients (age≥60 years). (**C**) Kaplan-Meier curves for patients with macroscopic disease ≤1cm. (**D**) Kaplan-Meier curves for patients with macroscopic disease >1cm. (**E**) Kaplan-Meier curves for patients with pathological grade 2. (**F**) Kaplan-Meier curves for patients with pathological grade 3. (**G**) Kaplan-Meier curves for patients with FIGO stage III. (**H**) Kaplan-Meier curves for patients with FIGO stage IV. FIGO, International Federation of Gynecology and Obstetrics.

To explore the impacts of clinical risk factors on the prognostic values of three-gene expression signature, a set of predefined subgroup analysis was conducted. We stratified the platinum sensitive patients from the validation group (n=161) by four risk factors, including age, residual disease, pathological grade and tumor stage (Table 2). Kaplan-Meier curves were conducted to visualize the survival probabilities for the low risk score versus high risk score group. We found that overall survival time of low risk score group was longer than high risk score group in patients with elder age [HR(95%CI) =2.39 (1.21-4.70); log-rank *P* value=0.006], high pathological grade [HR(95%CI) =2.06 (1.25-3.37); log-rank *P* value=0.002] and advanced FIGO stage [HR(95%CI) =1.65 (1.01-2.72) for stage III and HR(95%CI) =3.87 (1.46-10.3) for stage IV; all log-rank *P* value <0.05) (Figure 4A-H). In addition, we found the association between three-gene signature and overall survival was not affected by residual disease status (Figure 4C and 4D).

**Table 2:** Stratified analysis on the association between three-mRNA signature and OS of platinum-sensitive ovarian cancer patients in validating group.

**Variables**	**Total number**	**High risk score**			**Low risk score**		**HR (95%CI)**	***P* value**
		Case number	MST (month)		Case number	MST (month)		
Overall	161	81	49.7		80	67.2	2.08 (1.35-3.22)	0.001
Age (years)								
< 60	90	46	49.7		44	63.8	1.59 (0.90-2.82)	0.112
≥ 60	71	35	43.8		36	85.9	2.39 (1.21-4.70)	0.006
Residual Disease								
Macrospcopic disease ≤1cm	108	51	48.6		57	63.8	1.81 (1.08-3.34)	0.021
Macrospcopic disease >1cm	39	21	57.2		18	85.9	2.65 (1.12-6.29)	0.024
Pathological grade								
2	25	16	60.7		9	85.9	2.23 (0.69-7.25)	0.262
3	133	64	48.8		69	66.5	2.06 (1.25-3.37)	0.002
FIGO stage, no (%)								
III	128	63	51.8		65	63.8	1.65 (1.01-2.72)	0.043
IV	24	12	45.6		12	89.1	3.87 (1.46-10.3)	0.004

### Prediction of the three-gene signature associated biological pathways

**Figure 5:**
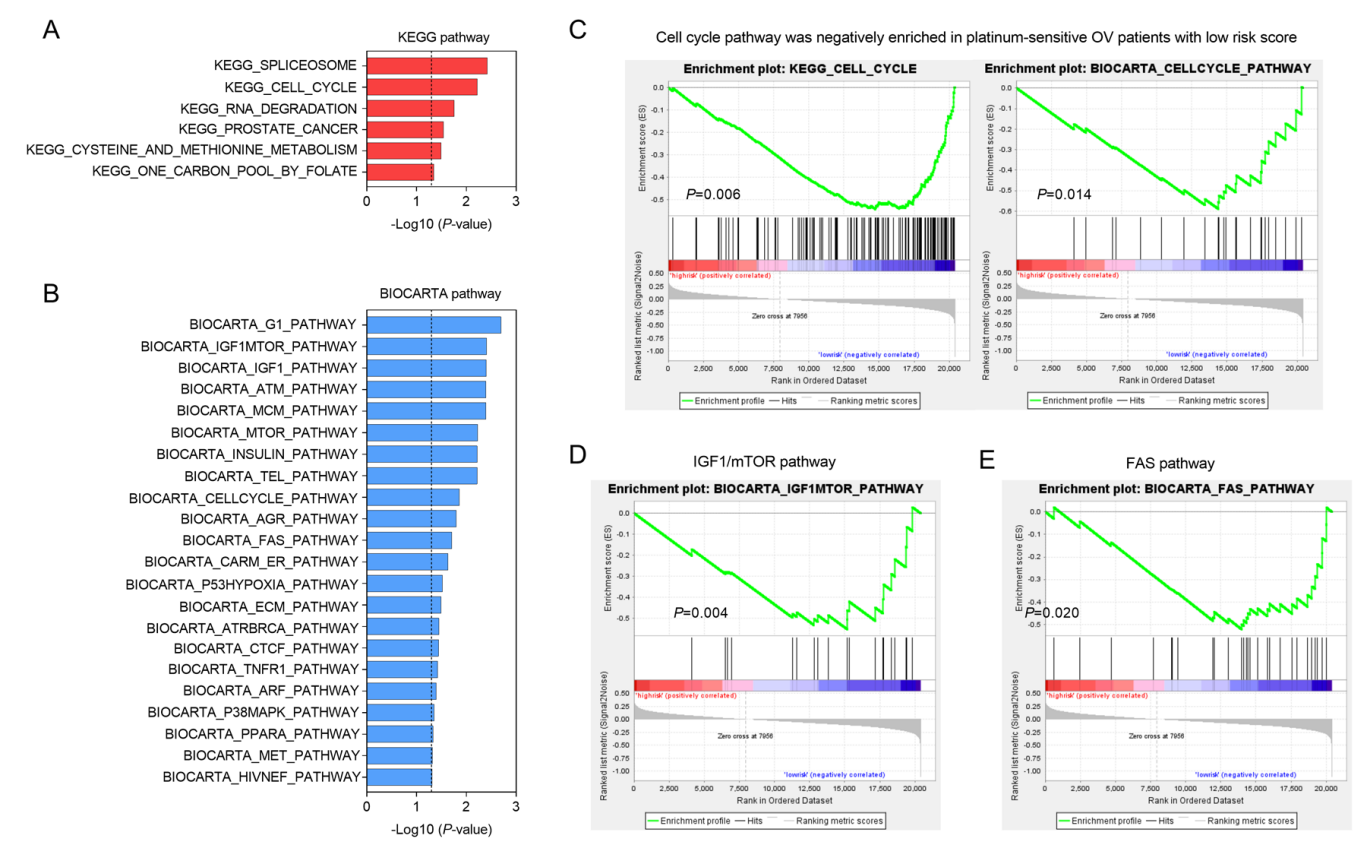
**GSEA delineates biological pathways associated with risk score in the validation group.** Significantly enriched KEGG pathways (**A**) and BIOCARTA pathways (**B**) of the co-expressed genes with three oxidative stress-related genes. GSEA validated downregulated activity of (**C**) cell cycle, (**D**) IGF1/mTOR and (**E**) Fas pathways in low risk score group.

To explore the biological processes and signaling pathways affected by the three-gene signature, we compared the genome-wide gene expression profile between high and low risk score groups in platinum sensitive patients by using GSEA. The significant KEGG and BIOCARTA gene sets were visualized as histogram bar charts. Six KEGG pathways and twenty-two BIOCARTA pathways were predicted to be correlated with three-gene signature (Figure 5A and 5B). Cell cycle pathway stood out in both of two gene sets, suggesting that the low risk score accompanied with down-regulation of cell cycle pathway (Figure 5C). In addition, two important signaling pathways,

IGF1/mTOR and Fas pathways, were also shown to be negatively enriched in platinum sensitive ovarian cancer patients with low risk score (Figure 5D and 5E). Above findings provided evidence for molecular mechanisms affected by three-gene signature in ovarian cancer patients received platinum-based chemotherapy.

## DISCUSSION

Ovarian cancer is the most common cancer with highest mortality rate among gynecologic cancers. Therefore, it is urgent to explore new prognostic biomarkers to predict the survival for patients with ovarian cancer. In this study, we firstly constructed a prognostic model consisting of a panel of three oxidative stress-related genes for ovarian cancer patients received platinum-based chemotherapy. Next, we evaluated the prognostic values of the three-gene signature in an independent group, and found that our risk model had high prognostic values in platinum sensitive patients. Finally, bioinformatic analysis suggested that the patients with low risk score was accompanied with down-regulation of cell cycle, IGF1/mTOR and Fas pathways.

For decades, researchers have found that oxidative stress-related genes are involved in cancer progression and therapeutic response. In ovarian cancer, genome-wide investigation also revealed that amount of oxidative stress-related genes were implicated in the carcinogenesis. Kajihara et al summarized the 54 highly up-regulated genes in ovarian cancer, and found 47 (87%) of them were redox-related genes, including oxidative and detoxification enzymes [16]. In the present study, we, for the first time, identified a panel of three oxidative stress-related genes, including *TXNRD1, GLA and GSTZ1,* to predict overall survival for ovarian cancer patients. These findings provide evidence for conducting a panel of oxidative stress-related genes as prognostic biomarkers in ovarian cancer.

*TXNRD1*, as a key regulation factor in oxidative stress control, was found to be associated with poor prognosis in breast cancer patients [17]. Saener Y et al identified four genes, including *TXNRD1*, were associated with clinical outcomes in patients treated with tremelimumab [18]. Recently, *TXNRD1* was found to be a risk factor for patients with hepatocellular carcinoma [19]. However, the prognostic value of *TXNRD1* in ovarian cancer has not yet been investigated. In our prognostic model, we identified *TXNRD1* as a risk factor for ovarian cancer patients. Moreover, we also found the patients with high risk scores had increased *TXNRD1* expression, consistent with the findings in other cancer types.

Glutathione S-transferases (GSTs) are a family of phase II isoenzymes that detoxify toxicant to lower toxic [20] and its dysfunction has been found to be closely related with response to chemotherapy [21–23]. *GSTZ1* belongs to the zeta class of GSTs, and patients carrying GSTZ1 variants had an increased risk of bladder cancer when exposed to trihalomethanes, a potential human carcinogen [24]. Mechanistic investigation suggested high levels of *GSTZ1* expression conferred resistance to the effect of anti-cancer therapy of dichloroacetate in hepatocellular carcinoma cell lines. In this study, we found *GSTZ1* might act as a protective factor in ovarian cancer, suggesting that altered *GSTZ1* expression level might have impact on survival by affecting the toxic of chemotherapy.

Moreover, in this study, we also found that several cancer-related pathways, such as cell cycle, IGF1/mTOR and Fas pathways, were related to three-gene signature. Cell cycle is a well-known critical factor that affects tumor progression. Many cell cycle regulators function as oncogenes that control proliferative and survival activities in chemo-response of ovarian cancer [7]. Our findings suggested that low risk score accompanied with down-regulation of cell cycle pathway, consistent with above knowledge. Moreover, we also found downregulation of IGF1/mTOR and Fas pathways in low risk score group. It has been have clearly demonstrated that IGF1/mTOR pathway took part in promoting cell proliferation [25] and affecting chemo-response [9,26] in ovarian cancer. Additionally, Fas protein was considered as a key factor mediating cell cycle and chemotherapy sensitivity [27]. These results implied important functional roles of the three-green signature in tumor progression and chemo-response of ovarian cancer patients.

In summary, using two independent cohorts and genome-wide gene expression profile, we systemically investigated the prognostic values of oxidative-stress related genes in ovarian cancer. We constructed a three-gene prognostic signature consisting of *TXNRD1, GLA and GSTZ1* which was associated with overall survival for ovarian cancer patients received platinum-based chemotherapy, especially in those with elder age, high pathological grade and advanced tumor stage. Further investigations are warranted to validate our findings.

## MATERIALS AND METHODS

### Sources of ovarian cancer patients

Two independent cohorts, AOCS (Australian Ovarian Cancer Study) and TCGA-OV (The Cancer Genome Atlas - Ovarian Cancer), were used in this study. The gene expression data of AOCS cohort (GSE9891) was downloaded from the Gene Expression Omnibus (GEO, http://www.ncbi.nlm.nih.gov/geo). GSE9891 consisted of 285 ovarian cancer samples and was performed on the Affymetrix Human Genome U133 Plus 2.0 platform. The gene expression data of TCGA-OV cohort was downloaded from the cBioPortal (http://www.cbioportal.org). TCGA-OV cohort consisted of 230 samples and was performed on the Illumina RNA-sequencing platform. All analyses were firstly conducted using the training dataset (GSE9891) and then validated using the validation dataset (TCGA-OV). Clinical characteristics of patients in the training and validation datasets were summarized in Table 3.

**Table 3:** Clinical features of ovarian cancer patients in the training and validating groups.

**Features**	**Training group** **(n=276)**	**Validating group** **(n=230)**
Age (years), (Mean±SD)	59.7±0.6	59.9±0.7
Residual Disease, no (%)		
No macropscopic disease	82 (29.7)	43 (18.7)
Macrospcopic disease ≤1cm	76 (27.5)	115 (50.0)
Macrospcopic disease >1cm	66 (23.9)	56 (24.3)
Unknown	52 (18.8)	16 (7.0)
Pathological grade, no (%)		
1	19 (6.9)	0 (0)
2	94 (34.1)	32 (13.9)
3	160 (58.0)	194 (84.3)
Unknown	3 (1.1)	4 (1.7)
FIGO stage, no (%)		
I+II	41 (14.9)	10 (4.3)
III	212 (76.8)	189 (82.2)
IV	22 (8.0)	31 (13.5)
Unknown	1 (0.4)	0 (0)
Progression status, no (%)		
Progression	33 (12.0)	197 (85.7)
Progression -free	106 (38.4)	33 (14.3)
Unknown	137 (49.6)	0 (0)
Vital status, no (%)		
Death	113 (40.9)	140 (60.9)
Alive	163 (59.1)	90 (39.1)

### Construction of prognostic signature

We screened the gene expression profile with the corresponding clinical data, and filtered out samples without clinical survival information. The therapeutic response to platinum was defined according to Liu’s method [15]. In brief, platinum-resistance was defined if tumor progress or recurrence within 6 months, and platinum-sensitivity was defined if the progression-free survival was more than 6 months. We then created the prognostic model, a risk-score formula, according to the expressions of candidate genes for survival prediction. Three oxidative stress-related genes, which were significantly and consistently associated with patients’ survival, were selected. Every patient was then accumulated a risk score that is a linear combination of the expression levels of the significant three genes weighted by their respective Cox regression coefficients. The risk score was calculated as follows: Risk score = (-0.38× expression value of *GLA*) + (-0.36 × expression value of *GSTZ1*) + (0.46 × expression value of *TXNRD1*).

### Survival analysis

Based on this risk score formula, patients in the training group were divided into low-risk and high-risk groups using the median value. The Kaplan-Meier curves were conducted to estimate survival time for the training and validation groups. Differences in median survival time (MST) between the low-risk and high-risk groups were then compared using the two-sided log rank test. Hazard ratio (HR) and 95% confidence intervals (CI) were calculated by Cox proportional hazards regression model.

### Gene set enrichment analysis (GSEA)

GSEA java software was downloaded from http://www.broadinstitute.org/gsea and analyzed using MSigDB C2 CP: BioCarta gene sets (217 gene sets available) and KEGG gene sets (186 gene sets available). Gene set with a *P*-value less than 0.05 was considered to be significantly enriched. Histogram bar charts and enrichment plots were used for visualization of the GSEA results.

### Statistical analysis

All data management and statistical analyses in the present study were conducted using R software with related packages (www.rproject.org). Categorical data was analyzed by Fisher’s exact test. The significance was defined as *P* values being less than 0.05.

## Supplementary Material

Supplementary File
