# Supplementary material for: A panel of three oxidative stress-related genes predicts overall survival in ovarian cancer patients received platinum-based chemotherapy
Source: Aging (Albany NY). 2018 Jun 17;10(6):1366–79. doi: 10.18632/aging.101473 (PMC6046245; doi:10.18632/aging.101473)
Supplement: Supplementary File [file aging-10-101473-s001.pdf]

SUPPLEMENTARY MATERIALS

Table S1. List of oxidative stress genes.

Symbol	Description	GeneBank
AKR1C2	Aldo-keto reductase family 1, member C2 (dihydrodiol dehydrogenase 2; bile acid binding protein; 3-alpha hydroxysteroid dehydrogenase, type III)	NM_001354
ALB	Albumin	NM_000477
ALOX12	Arachidonate 12-lipoxygenase	NM_000697
AOX1	Aldehyde oxidase 1	NM_001159
APOE	Apolipoprotein E	NM_000041
ATOX1	ATX1 antioxidant protein 1 homolog (yeast)	NM_004045
B2M	Beta-2-microglobulin	NM_004048
BAG2	BCL2-associated athanogene 2	NM_004282
BNIP3	BCL2/adenovirus E1B 19kDa interacting protein 3	NM_004052
CAT	Catalase	NM_001752
CCL5	Chemokine (C-C motif) ligand 5	NM_002985
CCS	Copper chaperone for superoxide dismutase	NM_005125
CYBB	Cytochrome b-245, beta polypeptide	NM_000397
CYGB	Cytoglobin	NM_134268
DHCR24	24-dehydrocholesterol reductase	NM_014762
DUOX1	Dual oxidase 1	NM_175940
DUOX2	Dual oxidase 2	NM_014080
DUSP1	Dual specificity phosphatase 1	NM_004417
EPHX2	Epoxide hydrolase 2, cytoplasmic	NM_001979
EPX	Eosinophil peroxidase	NM_000502
FHL2	Four and a half LIM domains 2	NM_001450
FOXM1	Forkhead box M1	NM_021953
FTH1	Ferritin, heavy polypeptide 1	NM_002032
GCLC	Glutamate-cysteine ligase, catalytic subunit	NM_001498
GCLM	Glutamate-cysteine ligase, modifier subunit	NM_002061
GLA	Galactosidase, alpha	NM_000169
GPX1	Glutathione peroxidase 1	NM_000581
GPX2	Glutathione peroxidase 2 (gastrointestinal)	NM_002083
GPX3	Glutathione peroxidase 3 (plasma)	NM_002084
GPX4	Glutathione peroxidase 4 (phospholipid hydroperoxidase)	NM_002085
GPX5	Glutathione peroxidase 5 (epididymal androgen-related protein)	NM_001509
GPX6	Glutathione peroxidase 6 (olfactory)	NM_182701
GPX7	Glutathione peroxidase 7	NM_015696
GSR	Glutathione reductase	NM_000637
GSS	Glutathione synthetase	NM_000178
GSTP1	Glutathione S-transferase pi 1	NM_000852

**Symbol**	**Description**	**GeneBank**
GSTZ1	Glutathione transferase zeta 1	NM_001513
GTF2I	General transcription factor IIi	NM_001518
HGDC	Human Genomic DNA Contamination	SA_00105
HMOX1	Heme oxygenase (decycling) 1	NM_002133
HPRT1	Hypoxanthine phosphoribosyltransferase 1	NM_000194
HSP90AA1	Heat shock protein 90kDa alpha (cytosolic), class A member 1	NM_001017963
HSPA1A	Heat shock 70kDa protein 1A	NM_005345
KRT1	Keratin 1	NM_006121
LHPP	Phospholysine phosphohistidine inorganic pyrophosphate phosphatase	NM_022126
LPO	Lactoperoxidase	NM_006151
MB	Myoglobin	NM_005368
MBL2	Mannose-binding lectin (protein C) 2, soluble	NM_000242
MGST3	Microsomal glutathione S-transferase 3	NM_004528
MPO	Myeloperoxidase	NM_000250
MPV17	MpV17 mitochondrial inner membrane protein	NM_002437
MSRA	Methionine sulfoxide reductase A	NM_012331
MT3	Metallothionein 3	NM_005954
NCF1	Neutrophil cytosolic factor 1	NM_000265
NCF2	Neutrophil cytosolic factor 2	NM_000433
NCOA7	Nuclear receptor coactivator 7	NM_181782
NOS2	Nitric oxide synthase 2, inducible	NM_000625
NOX4	NADPH oxidase 4	NM_016931
NOX5	NADPH oxidase, EF-hand calcium binding domain 5	NM_024505
NQO1	NAD(P)H dehydrogenase, quinone 1	NM_000903
NUDT1	Nudix (nucleoside diphosphate linked moiety X)-type motif 1	NM_002452
OXR1	Oxidation resistance 1	NM_181354
OXSR1	Oxidative-stress responsive 1	NM_005109
PDLIM1	PDZ and LIM domain 1	NM_020992
PNKP	Polynucleotide kinase 3'-phosphatase	NM_007254
PRDX1	Peroxiredoxin 1	NM_002574
PRDX2	Peroxiredoxin 2	NM_005809
PRDX3	Peroxiredoxin 3	NM_006793
PRDX4	Peroxiredoxin 4	NM_006406
PRDX5	Peroxiredoxin 5	NM_181652
PRDX6	Peroxiredoxin 6	NM_004905
PREX1	Phosphatidylinositol-3,4,5-trisphosphate-dependent Rac exchange factor 1	NM_020820
PRNP	Prion protein	NM_183079
PTGR1	Prostaglandin reductase 1	NM_012212
PTGS1	Prostaglandin-endoperoxide synthase 1 (prostaglandin G/H synthase and cyclooxygenase)	NM_000962
PTGS2	Prostaglandin-endoperoxide synthase 2 (prostaglandin G/H synthase and cyclooxygenase)	NM_000963

**Symbol**	**Description**	**GeneBank**
PXDN	Peroxidasin homolog (Drosophila)	NM_012293
RNF7	Ring finger protein 7	NM_014245
RPLP0	Ribosomal protein, large, P0	NM_001002
SCARA3	Scavenger receptor class A, member 3	NM_182826
SEPP1	Selenoprotein P, plasma, 1	NM_005410
SFTPD	Surfactant protein D	NM_003019
SIRT2	Sirtuin 2	NM_012237
SLC7A11	Solute carrier family 7 (anionic amino acid transporter light chain, xc- system), member 11	NM_014331
SOD1	Superoxide dismutase 1, soluble	NM_000454
SOD2	Superoxide dismutase 2, mitochondrial	NM_000636
SOD3	Superoxide dismutase 3, extracellular	NM_003102
SPINK1	Serine peptidase inhibitor, Kazal type 1	NM_003122
SQSTM1	Sequestosome 1	NM_003900
SRXN1	Sulfiredoxin 1	NM_080725
STK25	Serine/threonine kinase 25	NM_006374
TPO	Thyroid peroxidase	NM_000547
TRAPPC6A	Trafficking protein particle complex 6A	NM_024108
TTN	Titin	NM_003319
TXN	Thioredoxin	NM_003329
TXNRD1	Thioredoxin reductase 1	NM_003330
TXNRD2	Thioredoxin reductase 2	NM_006440
UCP2	Uncoupling protein 2 (mitochondrial, proton carrier)	NM_003355
VIMP	Selenoprotein S	NM_203472
